# Estimating the Number of Civilian Casualties in Modern Armed Conflicts–A Systematic Review

**DOI:** 10.3389/fpubh.2021.765261

**Published:** 2021-10-28

**Authors:** Amir Khorram-Manesh, Frederick M. Burkle, Krzysztof Goniewicz, Yohan Robinson

**Affiliations:** ^1^Institute of Clinical Sciences, Gothenburg University, Sahlgrenska Academy, Gothenburg, Sweden; ^2^Department of Research and Development, Armed Forces Center for Defense Medicine, Gothenburg, Sweden; ^3^Harvard Humanitarian Initiative, T.H. Chan School of Public Health, Harvard University, Boston, MA, United States; ^4^Department of Aviation Security, Military University of Aviation, Dȩblin, Poland

**Keywords:** armed conflicts, casualties, civilians, estimation, humanitarian law, tool

## Abstract

**Objective:** To examine the possibility of estimating the number of civilian casualties in modern armed conflicts.

**Methods:** A systematic review was conducted following the Preferred Reporting Items for Systematic Reviews and Meta-Analyses guidelines, using PubMed, Scopus, and Web of Science search engines. The outcome was analyzed using a qualitative inductive thematic analysis. The scientific evidence of selected article was assessed, using the Health Evidence Quality Assessment Tool.

**Findings:** The review of 66 included articles in this study indicates that with an increasing number of public health emergencies and the lack of vital elements of life such as water and food, emerging armed conflicts seem to be inevitable. In contrast to military-led cross-border traditional wars, modern armed conflicts affect internally on local communities and take civilian lives. Consequently, the measures and tools used in traditional military-led cross-border wars to adequately tally wounded and dead for many decades under the mandates of the International Humanitarian Law, is insufficient for modern warfare. While casualty counting during modern conflicts is deficient due to organizational, political or strategic reasons, the international organizations responsible for collecting such data (the International Federation of Red Cross and Red Crescent and International Institute of Humanitarian Law) face difficulties to access the conflict scene, resulting in under-reported, unreliable or no-reported data.

**Conclusion:** There are challenges in estimating and counting the number of civilian casualties in modern warfare. Although the global need for such data is evident, the risks and barriers to obtaining such data should be recognized, and the need for new international involvement in future armed conflicts should be emphasized.

## Introduction

The last decades' increase in globalization has contributed to an interdependency of the world's economies, cultures, technology, and populations through increased cross-border connectedness, exchange of information, trade, and mutual use of technique and routines (1, 2). This interdependency has further minimized perception of hostilities from other states, enhanced diplomatic agreements, military or economic alliances, resulting in an increased unwillingness of states to use military force unilaterally against another state and consequently a decline in the number of military-led cross-border traditional wars, hereinafter called Traditional Wars (3, 4).

While some may perceive globalization as an advancement for human society, which despite a need for continuous adjustment, promises new opportunities for all, others perceive globalization as a vehicle of economic and cultural disaster, and the main cause of the decline in the value of existing territorial interstate border, cultural and national identity (2, 5). The loss of identity and sense of belonging to a mutual goal results in the global rise of nationalism, and other types of reactionary movements, such as religious, and political, increased polarization in the society, and fosters extreme views and actions, leading to terrorism and modern armed conflicts (5–11).

Both traditional wars and modern armed conflicts have crucial impacts on society and result in material destruction and human losses. One way to evaluate the destructive outcomes of a war or an armed conflict is to estimate its impacts on human life (12–18). Knowing the utilized military means and strategies and their anatomical and physiological impacts on the human body along with registered data regarding the number and type of injuries from previous wars, the outcomes of a traditional war, in terms of mortality, morbidity, and the number of casualties has long been predictable, recorded, and debated (14–17). The information provides a reasonable ground for mental and practical preparedness before a war. This readiness has enabled estimation of needed resources for immediate assessment, treatment, and transport of victims, and has resulted in the development of military medicine, pre-hospital care, and mass casualty (injured and or killed) management (12, 13).

In contrast to traditional wars, modern armed conflicts involve networks of state and non-state actors with various means of military and militia influences and strategies. Such a combination creates difficulties in predicting the means and strategies associated with an armed conflict (9, 10, 12, 19). In addition, warfare in the 21st century constitutes multi-domain operations, asymmetry, and a hybrid approach (19–25). In hybrid warfare, the target of warfighting is not limited to the military staff and includes even civilians by creating political instability, conventional assaulting methods, riots, disinformation, influencing social media, and electoral outcomes (19, 21). Consequently, hybrid warfare may result in a larger number of civilian casualties not included in earlier estimation tools for traditional wars. The inability to estimate the casualty rate (16, 17) influences the calculation of needed resources, which creates a troublesome situation for the affected state and international help organizations. Particularly, the national healthcare contingencies organizations are dependent on predictions of medical support and resources needed to treat casualties (9, 10, 13, 26). Currently, casualty calculation relies mainly on registered and recorded data from earlier conflicts. While such data exist for traditional wars (14), it is missing in modern conflicts, reflecting the conflicting information regarding deaths and injuries from unreliable sources and conflicting estimations methods in modern conflicts (15–18).

With an increasing number of public health emergencies and the lack of vital elements of life such as water and food, emerging armed conflicts seem to be inevitable (26). This review aims to highlight the differences between traditional wars and modern conflicts, examine existing casualty estimation tools and evaluate the possibility of foreseeing the medical impacts of 21st-century warfare on the civilian population concerning the number of casualties, mortality, and morbidity.

## Methods

### Study Design, Searching Engines, and Searching Keywords

This systematic review was conducted following the Preferred Reporting Items for Systematic Reviews and Meta-Analyses (PRISMA) guidelines (27). According to PRISMA, the searching process yields an accumulated number of articles in the first step. In the next step, duplicates and non-relevant articles are removed. The abstracts of the remaining studies are studied to assure eligibility and relevance. A qualitative thematic analysis of the included literature based on an inductive approach is applied. This content analysis aims to study all included articles, focusing on similarities and differences in the findings to present the tentative results (28). Finally, each eligible article is thoroughly reviewed and the data, including the year of publication, author's name(s), the title of the study, and its scope are registered. The scientific evidence of each selected article is assessed, using the Health Evidence Quality Assessment Tool (Appendix 1), as Strong, Medium, or Weak (29). The initially designed electronic search model used PubMed, Scopus, and Web of Science to create a list of available literature in English, using the following search string: Armed conflict; Casualty estimation; Hybrid warfare; Mass casualties; Morbidity; Mortality; Traditional war; alone or in combination. The search was limited to literature in English and Russian language. The Swedish Defense University provided the latter.

### Inclusion and Exclusion Criteria

**Inclusions criteria:** Original publications and reviews dated January 2000 to July 2021.
**Exclusions criteria:** Proceedings, editorials, news, abstracts, and non-relevant papers.

### Ethical Approval

This study complied with the ethical principles stipulated by Swedish law. In Sweden, ethical approval is mandatory if the research includes sensitive data on the participants such as race, ethnic heritage, political views, religion, sexual habits, and health or physical interventions, or uses a method that aims to affect the person physically or psychologically. This study did not involve any human material or data regarding individuals and was based on available published data in scientific sources.

## Results

**Figure 1:**
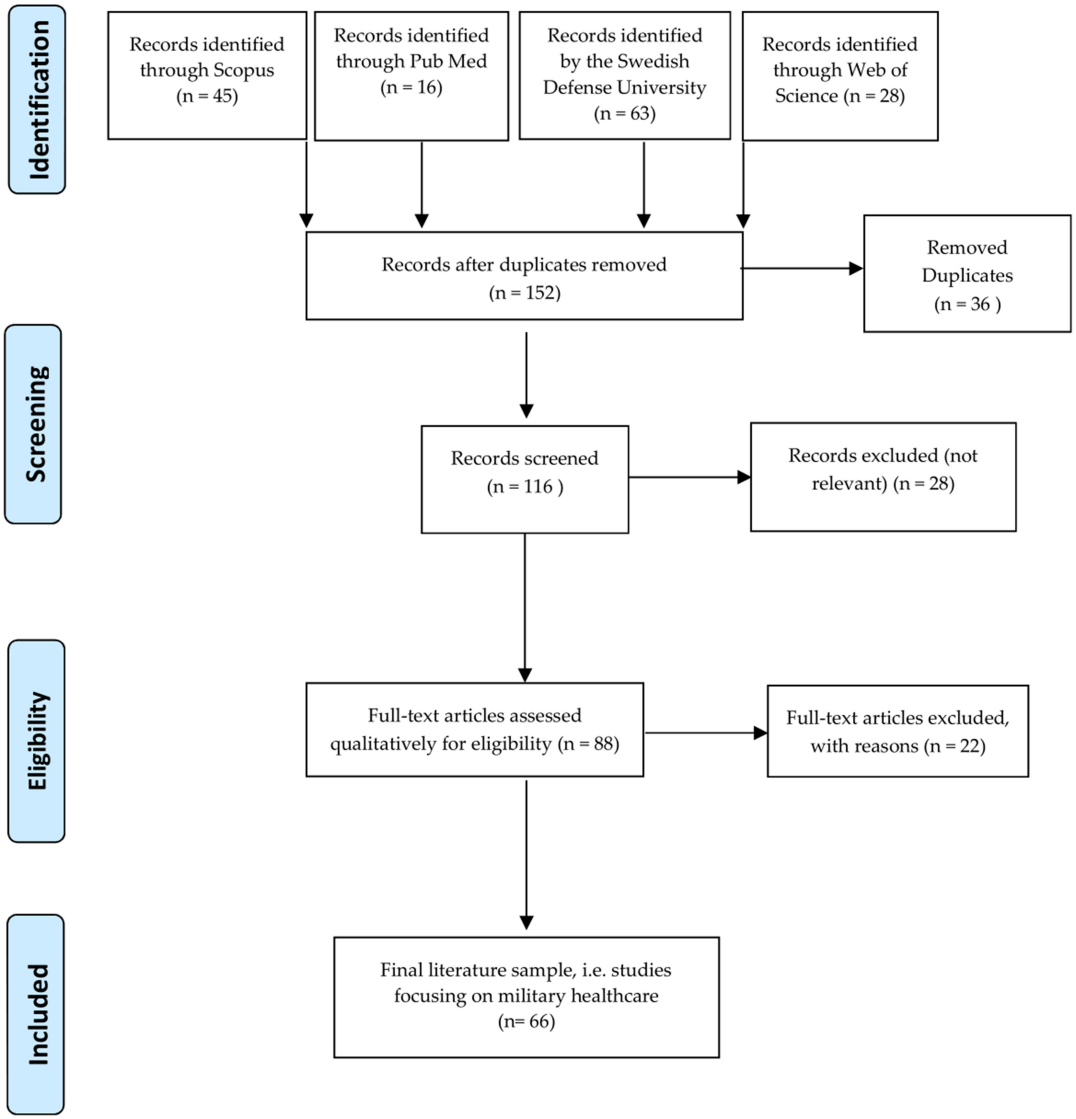
Flow diagram of included records for this study.

Initially, using “Armed Conflict” as the keyword, 136,476 publications were identified through the search engines. The number of hits was reduced to 2,501 by adding “Casualty Estimation,” and to 152, when all keywords were included. All included studies were reviewed. Special attention was paid to references within each eligible study that did not exist in the primary list to cover missed papers. The final list of publications was studied thoroughly and later included in the review (Figure 1). Summary of each paper, along with article information was transferred to a Microsoft Excel File and are presented in Appendix 2. Qualitative assessment of the included articles and content analysis allowed distinct categorization of the outcomes in diverse subgroups (see below). Articles categorized in each section were reviewed and relevant data were extracted.

### The Change of Paradigm in Warfare

There has been a change in warfare from military-led cross-border traditional wars (Traditional Wars), to those focused on local communities, and civilians (12, 19, 30). During Napoleon's wars, as well as other traditional wars, soldiers were the primary target and constituted the main group of casualties. Local civilians were warned, allowing them to flee their homes and hide to protect themselves from deaths and injuries (12). However, with advances in the arms industry, and changes in warfare strategies and ideologies over the last decades, the battlefields have moved into civilian's backyards, making them more vulnerable to and involved in wars (9). Consequently, there has been an increase in civilian fatalities from 5% at the turn of the 19th century to 15% during World War I (WW I), 65% by the end of World War II (WW II), and to more than 90% in the wars during 1990's, affecting more children than soldiers (12). During this period, there has also been a continuous blunder for the International Humanitarian Law (IHL) and Geneva Convention (GC) in favor of tactical and strategical harvesting, religious and political hatreds, the collapse of State structures, mastering the scarcity of natural resources, the vast availability of weapons, increased acts of terrorism and the spread of asymmetric conflicts (12, 31). Table 1 presents the available minimum reported number of totals, military, and civilian deaths in some major wars and armed conflicts (1950–2019), demonstrating the changing paradigm of warfare from the traditional wars to locally-focused armed conflicts (32–35). The civilian death ratio obtained by dividing civilian deaths by the total number of deaths aims to compare the outcome of the various war and to indicate a possible trend.

**Table 1:** The outcome of wars/conflicts in terms of mortality.

**Major Military Conflicts**						
**Conflict**	**Period**	**Total deaths**	**Military deaths**	**Main foreign army**	**Civilian deaths**	**Civilian death rate**
Korean War	1950–1953	2,238,172	579,736	33,686	1,658,436	74%
Vietnam War	1965–1974	1,353,000	726,000	58,200	627,000	46%
Persian Gulf War	1990–1991	162,341–232,541	20,341–26,541	341	142,000–206,000	87%−88%
Balkan War	1991–2001	130,000–140,000	-	-	72,716	52%−56%
2nd intifada	2000–2007	5,848	2000	-	3000	51%
Afghanistan	2001–2019	157,052	113,481	2,298	43,571	28%
Pakistan	2001–2019	66,063	41,956	0	24,107	36%
Iraq	2003–2019	276,363–308,212	91,626–100,701	4,572	184,737–207,511	66%−67%
Syria/ISIS	2014–2019	179,424	129,572	7	49,852	28%
Yemen	2002–2019	90,072	78,003	1	12,069	13%
Ukrainian	2014–2019	13,117–13,496	9,750–10,129	500	3,367	25–26%

*Conflicts with unreliable or missing civilian death numbers are not included, e.g., Yom Kippur, Chechnya, and Iran-Iraq war. (- = Not available)*.

Besides the paradigm change, the available data demonstrates a variation in the rate of civilian deaths. Shorter and probably more intensive wars seem to be associated with a higher number of civilian deaths, while recent long-term conflicts show a lower rate of civilian deaths, a decline in the number of military deaths, as well as, a decrease in the number of foreign military service members participating in different conflicts. The latter might be indicative of an increasing number of proxy wars (16, 17).

### The Reliability of Data Regarding Deaths and Injuries

Reporting the correct number of deaths and casualties is required for the selection of necessary measures to avoid human catastrophe. In agreement with other published data, Tables 1, 2 show the varying estimate for death tolls presented in this review (36, 37). Although some variations, the casualty reports concerning terror attacks, presented in Table 2, seem to be more reliable than the one from modern armed conflicts (Table 2). These differences may indicate the diversified information obtained in each conflict due to differences in analyzing methods, or other reasons such as:

**Table 2:** The outcome of some major terror attacks in terms of mortality and morbidity.

**Major Terror Attacks**					
**Attack**	**Year**	**Number of deaths**	**Number of injured**	**Ratio deaths/Total casualty**	**Means of terror**
Oklahoma City	1995	167	759	18%	Explosion
New York	2001	2,996	6,000	49%	Explosion
Madrid	2004	191	2,000	9%	Explosions
London	2005	56	775	7%	Explosions
Boston	2013	3	281	1%	Explosions
Paris	2015	129	300	30%	Explosions
Brussels	2016	35	300	10%	Suicide Bombing
Las Vegas	2017	58	600	8%	Gunshot
Sri Lanka	2019	279	593	32%	Suicide Bombing
Christchurch	2019	51	49	51%	Gunshot

First, the traditional wars symbolize an armed disagreement between two or several countries, normally following the International Humanitarian Law (IHL) (12, 38). IHL is a set of rules, which seek humanitarian reasons, to limit the suffering, losses, and other effects of armed conflict by restricting the means and methods of warfare to protect individuals who are not or are no longer participating in the hostilities (30). According to IHL, “A State” has the responsibility for all attributable violations of IHL committed by its organs (including its armed forces), and persons or entities it empowered to exercise elements of governmental authority. It is also responsible for the deeds of those acting in fact on its instructions, or under its direction or control, and by private persons or groups, which it acknowledges and adopts as its conduct (30, 38, 39). In contrast to traditional wars, there is constant negligence of IHL and GC implementation in modern armed conflict. It is simply much more important to achieve the tactical and strategical goal in a conflict than saving civilian lives (12, 30).

Moreover, three international bodies are mainly involved in the development and implementation of IHL. The International Federation of Red Cross and Red Crescent Movement (IFRC), and the International Institute of Humanitarian Law (IIHL). The former is one of the three components of the International Movement, which besides IFRC, also consists of the International Committee of the Red Cross (ICRC), and Red Cross Red Crescent National Societies. ICRC is an operational institution that protects victims of conflicts within a country as well as across boundaries, while IFRC is the largest humanitarian organization. The IFRC coordinates between National Red Cross and Red Crescent Societies globally and along with ICRC supports the foundation of new National Societies in countries where no official society exists. A National Society becomes a member to the IFRC only after the ICRC recognizes it. These organizations work in close collaboration with other international organizations dedicated to the humanitarian cause, such as the United Nations High Commissioner for Refugees (UNHCR), and have operational relations with the European Union (EU), North Atlantic Treaty Organization (NATO), and others (38–40). According to IHL, ICRC have the mandates to gain insights into an ongoing conflict, and as an impartial, neutral, and independent organization protect the lives and dignity of victims of war and internal violence. They assist the affected population, direct and coordinate the international relief activities, promote the importance of IHL, and draws attention to universal humanitarian principles (40, 41). Additionally, they also have mandates to visit prisons, organize relief operations, reunite separated families, meet the needs of internally displaced persons, raise public awareness of the dangers of mines and explosive remnants of war, and trace people who have gone missing during conflicts (40). All these tasks give ICRC a possibility to track war activities and to present a real picture of the war in terms of the numbers of casualties and deaths. In contrast to traditional wars, modern armed conflicts involve networks of state and non-state actors with various means of military and militia influences and strategies. Such a combination creates difficulties in the implementation, control, and evaluation of IHL's “A State” responsibility principle and does not allow international organizations to get an insight into an armed conflict (9, 10, 12, 30).

Furthermore, casualty recording is a systematic and continuous process of documenting individual direct deaths from armed conflict or widespread violence (42), which is normally conducted by public services normally involved in recording deaths, such as hospitals, coroners, and police forces, within determined scope, usually bound by time and location. While the internal structure of states can be intact in a majority of traditional wars (may not apply to invaded nations), these entities are no longer functioning effectively in many armed conflicts. In contrast to traditional wars, modern armed conflicts target the local structures, organizations, and public services and aim at destabilizing the authorities and societal networks (13, 30, 31, 41, 43–45). Warfare in the 21st century constitutes multi-domain operations, asymmetry, and a hybrid approach (19–22). In hybrid warfare, the target of warfighting is not limited to the military staff and includes even civilians and the social structure of a nation by creating political instability, conventional assaulting methods, riots, disinformation, influencing social media, and electoral outcomes. Consequently, hybrid warfare leads to a society in chaos with no functional entities. In such a society, the ordinary sources of reporting do not exist and the reliability of information and information sources are questionable. Thus, influencing the calculation of needed resources and creating a troublesome situation for the affected state and international help organizations. Particularly, the healthcare system, which fails to predict the medical support and resources needed to treat casualties (9, 10, 23–25, 30).

Finally, there might be political reasons why state authorities do not publish or share information on conflict-related deaths or might create a different definition for a civilian casualty. Several nations in conflicts are governed by autocratic regimes, which have failed to adopt investments in public health infrastructure, education, and prevention measures to keep pace with population growth and density (41, 46). These nations have leaders that do not understand the impact and consequences of war and armed conflicts, as well as, other disasters and emergencies on their population. They directly influence health security, and structure and create situations with adverse political and economic outcomes that only complicate the crisis further (41). Consequently, in the absence of official recording processes, casualty recording is frequently conducted by civil society organizations, or some internationally mandated entities, such as United Nations peacekeeping missions (42, 47, 48). While these organizations might be widely present in traditional wars, their presence in modern armed conflicts is limited or non-existing, resulting in conflicted and unreliable reports from other sources.

## Current Casualty and Deaths Statistic

### Interstate Wars

Compared to WW II (Table 1), the number of civilian deaths and injuries caused by rockets and bombs decreased in Korean War, while the mortality caused by grenades, land mines, and other fragmentary explosions increased in both civilian and military populations (34, 35, 49, 50). Consequently, the civilian death rate in Vietnam decreased to 46% compared to that of 74% in the Korean War (51). The former lasted 3 years, and the latter is around 10 years. During the first Persian Gulf War, the civilian death rate increased to 87–88% with a variation in the number of civilian deaths and an undefined number of injuries (43, 52, 53). The multiple ethnical wars in Former Yugoslavia (1991–2001), on the other hand, lasted almost 10 years and resulted in lower civilian death rates of 52–56% (54–56). Estimates of civilian casualties from the Israeli–Palestinian conflict differs both in numbers and sources, however, the United Nations Office for the Coordination of Humanitarian Affairs (OCHA) reported a civilian death rate of 51% from the beginning of the second intifada in September 2000 until the end of July 2007 (47).

The still ongoing conflicts in Afghanistan, Syria, Pakistan, Iraq, and Yemen represent armed conflicts, which engage several countries, militant groups, and strategies. There are contradictory reports of civilian death rates from these conflicts ranging from 28% in Afghanistan, 36% in Pakistan, 67% in Iraq, 28% in Syria, and 13% in Yemen (34, 57–59). Although the civilian casualty ratio for drone strikes is notoriously difficult to quantify, the U.S. estimates a very low number of civilians killed from its drone strikes in Pakistan. A recent study found non-militant casualty rates starting high but declining steeply over time, from about 60% (3 out of 5) in 2004–2007 to <2% (1 out of 50) in 2012. The study puts the overall non-militant casualty rate since 2004 at 15–16% (59).

A few reports are available from Russian's involvement in foreign missions. The estimated number of deaths and injuries during Russian foreign missions are around 17,453 deaths and 471,406 injuries from 1946 to 1989. The total numbers reported for 1901–1999, including world wars are 12,132,668 deaths and 35,669,180 injuries. Only during 1939–1945, were 3,392 deaths and 8,738 injuries per day reported. Although high, the reported figures for WWI (1914–1918), and the Russian civil war (1918–1922) are slightly lower. It is not clear how many of these were military service members and how many civilians (60, 61). The Chechen Wars resulted in a large number of civilian deaths. According to Russian sources, the number of deaths and injuries in the first Chechen war was 3,927, and 17,892, respectively. The number of injured in the second Chechen war is missing but 3,669 were dead. There is no information about civilian deaths and injuries (61–63).

The recent Ukrainian armed conflict, which started in March 2014, and in the aftermath of the 2014 Ukrainian revolution, has engaged Russia-backed anti-government separatist groups and Ukrainian Army, National Guard, and voluntary organizations. The conflict has all ingredients of a proxy and hybrid war, in which different parts claim superiority over the others with high impacts on civilian life. The United Nations (UN) reports over 13,000 deaths from April 2014 to February 2020. The number of civilian's deaths reported is 3,367 from April 2014 to July 2020 (civilian death rate = 26%). The reported number of military and voluntary forces deaths are conflicting (64–67).

Finally, the recent Nagorno-Karabakh conflict between ethnic Armenian and Azerbaijani armies has resulted in many casualties and deaths with both sides downplaying the number of their casualties and exaggerating the numbers of enemy casualties and injuries (68).

### Terror and Internal Conflicts–a Part of Future Hybrid Warfare

The data demonstrated in Table 2 concerning some recent terror attacks, indicates the new wave of internal conflicts and terror. The target of these attacks is the local communities and civilians. The intensity and severity of attacks are diverse and the number of deaths and injured diversified. The majority of cases represent political and religious motives. Explosives and suicide bombings have been the main means of terror. Almost all injured and deaths are civilians, with a variation of death numbers from 3 to as much as 2,996, and injured from 49 to 6,000, and a ratio of deaths/total casualty of 8 to 51% (69–77). One important denominator of these attacks is the chaos and overwhelming pressure they created for emergency services, particularly healthcare. The number of deaths is not an immediate concern, however, a high number of injuries require both a multiagency approach and availability of healthcare in several hospitals and healthcare facilities, along with a local preparedness at the community level for both adult and pediatric conditions and military-like injuries.

### Calculating Civilian Casualty in Modern Armed Conflicts

In this review, the rate of civilian mortality varies from 13 to 87%. Previous studies have reported a civilian casualty rate of 65 to 70% of the total casualties in a war (13, 32–35, 49). The number of deaths and injuries in the future modern armed conflicts can only be assessed hypothetically since each conflict has its characteristics. However, as shown in this study, the number of civilian casualties will still be high and might be comparable with that of wars in the former Yugoslavia, and Syrian (54–56). The figures from the domestic conflicts do not influence the total number of civilian casualties and deaths markedly. However, its significance lies in the fact that multi-level confrontations and assaults result in resource scarcity, particularly within the healthcare systems, over a longer period, causing a rise in the number of deaths, and a need for serious medical decision-making (5, 9, 13, 44, 78–82).

Assuming that the number of deaths is a technical problem, the overwhelming number of injuries will be the cause of the collapse in all systems (11, 19, 83). The 90% increase in the global urban population in developing countries over the next two decades increases these nations' vulnerability to political and social unrest, violent crimes, terrorism, disasters, and armed conflicts (81, 84). However, previous estimation algorithms, such as the one introduced by Kuhn used for traditional wars (14), fail to estimate the casualty and mortality numbers of future armed conflicts.

## Discussion

The aims of this review were to highlight the differences between traditional wars and modern conflicts, investigate existing casualty estimation tools and evaluate the possibility of foreseeing the medical impacts of 21st-century warfare on the civilian population concerning the number of casualties, mortality, and morbidity. Although this study fails to find a simple algorithm to estimate civilian casualties, it outlines a change in warfare paradigm from traditional wars to modern, locally-focused conflicts (11, 18, 19). Furthermore, it recognizes the involvement of a larger portion of civilians in modern conflicts and consequently a large number of casualties that the ordinary healthcare system may not be able to manage, with or without a reliable and modern casualty estimation tool (15–17). Finally, it also highlights the continuous negligence of the International Humanitarian Law and Geneva Conventions in the recent conflicts, which not only prevent the mandated international organization to surveil the modern conflicts but also threatens the democracy and well-being of a world exposed to continuous change and emerging hazards (12, 26, 41).

Although, efficient and appropriate estimation of the number of deaths and wounded is a necessary part of mass casualty management, it remains challenging in both civilian and military settings due to several decisive factors (12, 15–17, 34, 35, 49, 57, 84, 85):

a) The maximum capacity of each system: There is always a limit on how expandable a system is due to financing and available resources?
b) The shape and condition of the infrastructure: There is always a limit on how many facilities can be used and if the transport routes to these facilities are intact?
c) The grade of preparedness (resilience and resources): Are all entities, including communities, prepared, and is the collaborative element of preparedness exercised and trained?
d) The etiology and cause of mass casualty including the weaponry used: Chemical, Biological, Radiological, Nuclear, or Explosives. Terrorism or interstate invasion?
e) The incident's (combat) size and intensity: Large or small area, long- or short-term?
f) The demography and density of the population involved. Populations background concerning aggressors. High- or low-populated areas?

The slow transition of traditional wars with mainly military engagement to very different modern conflicts, engaging civilians has not only brought about a change in warfare paradigm but also an increase in the number of civilian casualties (11, 12, 19, 34, 35, 49, 78–80, 85). The current state of globalization and the technological advancement within the arms industry enable nations to avoid interstate conflict and direct involvement, using proxy fighters and escaping state's responsibility in following the International Humanitarian Law and Geneva Convention (24, 25, 30, 85, 86).

The collection of data under the IHL mandates by ICRC, among others, facilitated the necessary information to limit and guide the use of weaponry and to protect civilians, and assist both sides of the war. While the use of the casualty estimation tools in traditional wars enabled the tally and management of the casualties, it did not include the count of civilians since the rules of wars were different (12, 30, 40, 44). The use of new technology and the development in the weapon industry and safety items such as body armor has resulted in a decline in the number of casualties caused by direct fire and small arms injuries. Furthermore, the development of trauma care and evacuation option has also resulted in fewer injuries and deaths on the military side (34, 35, 79–81, 87–91). Nevertheless, these successes have also resulted in the development of isolated, local, and urban conflicts, high rates of explosions, and close encounters, influencing the civilian population.

New military strategies, remote warfare, and the use of drones, proxy fighters, and hybrid warfare, present the face of modern and unconventional warfare, which not only threatens and takes civilian lives, but also raises new ethical and moral concerns when violating IHL and GC (12, 30, 81, 82). Additionally, modern conflicts generate millions of displaced persons, which overwhelms the capacity of healthcare and involved relief organizations (26, 64–66, 80–95). Such development increases the vulnerability of protective authorities, consumes legal and healthcare systems, paralyzes the national government and finally may dissolve national unity (9, 25, 30, 45). These are all factors that enhance the violation of human right and equality with no punishment. These scenarios endanger the mandated work of international organizations to supervise and regulate the rules of the war. It also disables the possibility of receiving correct information and enhance the possibility of belligerents and terrorist to hamper a democracy. This is certainly a global and unique problem for organizations such as ICRC, which have the responsibility to gather data and collect the necessary information to save civilian lives under IHL, and calls for actions targeting countries or warring factions that do not recognize the rules of war.

In this review, the rate of civilian casualties varied from 13 to 87% of the total casualties, depending on the type of conflict and might be concordant to that reported from earlier reports regarding wars in the literature (65–70%) (34, 35, 49–51). It is, however, clear that even 13% of the population involved in a little conflict, such as the one in Ukraine or Nagorno-Karabach, can result in over thousands injured, which alone can paralyze any local healthcare system. Together with the injuries from internal violence, riots, and assaults, the accumulated population in need of emergency help can overtime be comparable to the wars in the former Yugoslavia, and Syrian (47, 54–56, 58). The number of deaths, according to the same estimation, may vary up to 30% of the injured (32–35, 49). The figures from the domestic conflicts do not influence the total number of civilian casualties and deaths markedly. However, its significance lies in the fact that multi-level confrontations and assaults result in resource scarcity, particularly within the healthcare systems, over a longer period, causing a rise in the number of deaths, and a need for serious medical decision-making (9–11, 18, 33, 34, 45, 96–98). Assuming that the number of deaths is a technical problem, the overwhelming number of injuries will be the cause of the collapse in all systems, requiring multiagency collaboration and a flexible surge capacity (96–98). The 90% increase in the global urban population in developing countries over the next two decades increases these nations' vulnerability to political and social unrest, violent crimes, terrorism, disasters, and armed conflicts (9, 10, 84). Medical planning for modern armed conflicts in the future should include an estimation of casualties in urban areas caused by domestic assaults, the use of drones, and terror attacks.

## Limitations

The presented analysis has limitations.

- The majority of publications used in this review are in English, except a few translated Russian references. Consequently, some interesting data in other languages might be missing.
- Appropriate estimation of the casualties relies on complete data. There has been missing data regarding: The number of injuries and deaths among civilians and military staff, and some of the estimations were not reliable to use. Some major wars, such as the war between Iran and Iraq, were not included due to the lack of final data from the Iraqi side.
- There is a lack of standard definition of civilian casualty caused by armed conflicts.
- Additionally, there was neither complete information about casualties of air raids, nor the use of CBRN (Chemical, Biological, Radiological, Nuclear). Conflicts may also lead to the displacement of large populations into temporary settlements or camps with overcrowding and rudimentary shelters, inadequate safe water and sanitation, and increased exposure to disease vectors during the acute phase of the emergency. Thus, no available casualty figures for such incidents.
- In protracted and post-conflict situations, populations may have high rates of illness and mortality due to breakdown of health systems, flight of trained staff, failure of existing disease control programs, and destroyed infrastructure. These populations may be more vulnerable to infection and disease because of high levels of undernutrition or malnutrition, low vaccine coverage, or long-term stress.
- Finally, more and more defense policies identify the cyber (or information) domain and the human domain as to be included in multi-domain warfare (99, 100). Since warfare in cyber and human domains involve substantial pillars of civilian society, civilian contingencies planning requires casualty estimation in these warfare. Currently, the civilian and military casualty rates from cyber and human domain warfare is unavailable and could not be included in this review.

## Conclusion

With an increasing number of public health emergencies and the lack of vital elements of life such as water and food, emerging armed conflicts seem to be inevitable (20, 87). This creates a unique and crucial situation in need of resource assessment and planning, which in turn requires a detailed study on the cause and impacts of the modern armed conflicts and clear access to the fields for supervision of the outcomes, casualty recording, and rules of the war (30, 45, 101, 102). The undeniable failure of international bodies to commit to humanitarian principles and the global disarray of the humanitarian system indicates the need for extensive reform in the current structure or a new global humanitarian body. Such afresh organization needs to employ a decentralized model to manage aid funds, assume coordination of international responses, resolve civil-military coordination, cater for people affected by both conflict and disasters, and professionalize the humanitarian career (101–103). Meanwhile, using the data presented in this study, even the lowest number of casualties inflicted by the modern armed conflicts may be enough to paralyze any healthcare system and indicates a need for new measures beyond a simple casualty estimation tool. Enhancing multiagency collaboration, empowering local preparedness and resilience capacity, and creating a flexible surge capacity might be new approaches, which together with a new international governing structure can achieve a better future for the next generation (30, 96–98).

This review aimed to discuss and examine the outcomes of traditional wars and modern armed conflicts and the possibility of foreseeing the medical impacts of 21st-century warfare on the civilian population concerning the number of casualties, mortality, and morbidity. Although it fails to present a simple casualty estimation algorithm, it highlighted the need for international engagement and each state's responsibility in following the rules of war. However, the most important factor remains to be the increased understanding of the nature of modern warfare and to plan for a scenario, when the needs exceed available resources, and decisive triage and adjusted resource utilization are mandatory.

## Recommendations

1. The management of modern armed conflicts needs resources beyond casualty estimation tools. Multiagency collaboration, risk and vulnerability analysis and a high level of preparedness may improve the response in all phases of wars/conflicts.
2. Strengthening the international engagement, the role of humanitarian organizations, and the gravity of the International Humanitarian Law and Geneva Convention plays a crucial role in future conflicts. A new or a re-organized international governing agency is needed to hold all nations responsible for their actions by issuing, implementing, and supervising new restrictive approaches and international legal standards to production, and utilization of new weapons, and strategies. Furthermore, new strategies should be developed to combat new trends, such as the process of delegating the performance of traditional state functions by states in favor of private military and security companies.
3. Empowering the local preparedness, risk reduction and resilience facilitates a proper response at the local level before additional resources can be obtained. Such readiness requires a functional public health and public service and an investment in education of local population. The cost will be far less than what the conflicts in general and modern armed conflict, in particular, may generate.

## Data Availability Statement

The original contributions presented in the study are included in the article/Supplementary Material, further inquiries can be directed to the corresponding authors.

## Author Contributions

AK-M provided the main framework, identified, and organized primary materials, and collaborated in writing the manuscript. FB reviewed and collaborated on the writing of the manuscript. KG and YR contributed to drafting sections of the manuscript. All authors have read and agreed to the published version of the manuscript.

## Conflict of Interest

The authors declare that the research was conducted in the absence of any commercial or financial relationships that could be construed as a potential conflict of interest.

## Publisher's Note

All claims expressed in this article are solely those of the authors and do not necessarily represent those of their affiliated organizations, or those of the publisher, the editors and the reviewers. Any product that may be evaluated in this article, or claim that may be made by its manufacturer, is not guaranteed or endorsed by the publisher.
