# Supplementary material for: Estimating the Number of Civilian Casualties in Modern Armed Conflicts–A Systematic Review
Source: Front Public Health. 2021 Oct 28;9:765261. doi: 10.3389/fpubh.2021.765261 (PMC8581199; doi:10.3389/fpubh.2021.765261)
Supplement: Supplementary file 1 [file Data_Sheet_1.PDF]

Appendix 1

Health Evidence™

Helping public health use best evidence in practice since 2005

Quality Assessment Tool – Review Articles

Instructions for completion:

Please refer to the attached dictionary for definition of terms and instructions for completing each section. For each criteria, score by placing a check mark in the appropriate box.

First Author: ______________________
Year: ______________________
Journal: ______________________
Reviewer: ______________________

CRITERIA	YES	NO
Q1. Did the authors have a clearly focused question [population, intervention (strategy), and outcome(s)]?		
Q2. Were appropriate inclusion criteria used to select primary studies?		
Q3. Did the authors describe a search strategy that was comprehensive?<br>*Circle all strategies used*:<br>▪ health databases<br>▪ psychological databases<br>▪ social science databases<br>▪ educational databases<br>▪ other<br>▪ handsearching<br>▪ key informants<br>▪ reference lists<br>▪ unpublished		
Q4. Did search strategy cover an adequate number of years?		
Q5. Did the authors describe the level of evidence in the primary studies included in the review?<br>▪ Level I → RCTs only<br>▪ Level II → non-randomized, cohort, case-control<br>▪ Level III → uncontrolled studies		
Q6. Did the review assess the methodological quality of the primary studies, including:<br>*(Minimum requirement: **4/7** of the following)*<br>▪ Research design<br>▪ Study sample<br>▪ Participation rates<br>▪ Sources of bias (confounders, respondent bias)<br>▪ Data collection (measurement of independent/dependent variables)<br>▪ Follow-up/attrition rates<br>▪ Data analysis		
Q7. Are the results of the review transparent?		
Q8. Was it appropriate to combine the findings of results across studies?		
Q9. Were appropriate methods used for combining or comparing results across studies?		
Q10. Do the data support the author’s interpretation?		
TOTAL SCORE:		

Quality Assessment Rating:	Strong (total score 8 – 10)	Moderate (total score 5 – 7)	Weak (total score 4 or less)
